# Wogonin ameliorates the proliferation, inflammatory response, and pyroptosis in keratinocytes via NOD‐like receptor family pyrin domain containing 3/Caspase‐1/Gasdermin‐D pathway

**DOI:** 10.1002/iid3.1303

**Published:** 2024-07-05

**Authors:** Jun Ma, Chen Ji, Yanhong Sun, Danqing Liu, Kai Pan, Yuegang Wei

**Affiliations:** ^1^ First College of Clinical Medicine Nanjing University of Chinese Medicine Nanjing China; ^2^ Department of Dermatology The Affiliated Zhangjiagang Hospital of Soochow University Suzhou China; ^3^ Department of Dermatology Zhangjiagang TCM Hospital Affiliated to Nanjing University of Chinese Medicine Suzhou China

**Keywords:** inflammatory response, NOD‐like receptor family pyrin domain containing 3/Caspase‐1/Gasdermin‐D pathway, psoriasis, pyroptosis, Wogonin

## Abstract

**Background:**

Psoriasis refers to a highly prevalent and immunologically mediated dermatosis with considerable deterioration in life quality. Wogonin, a sort of flavonoid, has been mentioned to elicit protective activities in skin diseases. However, whether Wogonin is implicated in the treatment of psoriasis and its specific mechanisms are not fully understood.

**Aim:**

The present work attempted to elaborate the role of Wogonin during the process of psoriasis and to concentrate on the associated action mechanism.

**Methods:**

Cell counting kit‐8 (CCK‐8) method was initially applied to assay the viability of human keratinocyte HaCaT cells treated by varying concentrations of Wogonin. To mimic psoriasis in vitro, HaCaT cells were exposed to M5 cytokines. CCK‐8 and 5‐Ethynyl‐2′‐deoxyuridine  assays were adopted for the measurement of cell proliferation. Inflammatory levels were examined with enzyme‐linked immunosorbent assay. Immunofluorescence staining tested nucleotide‐binding oligomerization domain (NOD)‐like receptor family pyrin domain containing 3 (NLRP3) and Caspase‐1 expressions. Western blot examined the protein expressions of proliferation‐, inflammation‐, pyroptosis‐associated factors, and NLRP3.

**Results:**

Wogonin treatment antagonized the proliferation, inflammatory response, and NLRP3/caspase‐1/Gasdermin‐D (GSDMD)‐mediated pyroptosis in M5‐challenged HaCaT cells. Besides, NLRP3 elevation partially abrogated the effects of Wogonin on M5‐induced proliferation, inflammatory response, and NLRP3/caspase‐1/GSDMD‐mediated pyroptosis in HaCaT cells.

**Conclusion:**

In a word, Wogonin might exert anti‐proliferation, anti‐inflammatory and anti‐pyroptosis activities in M5‐induced cell model of psoriasis and the blockade of NLRP3/Caspase‐1/GSDMD pathway might be recognized as a potential mechanism underlying the protective mechanism of Wogonin in psoriasis, suggesting Wogonin as a prospective anti‐psoriasis drug.

## Graphical abstract

Wogonin was capable of alleviating the hyperproliferation, inflammatory response as well as pyroptosis in HaCaT keratinocytes exposed to M5 cytokines, the effects of which might be related to the blockade of NOD‐like receptor family pyrin domain containing 3/Caspase‐1/Gasdermin‐D  pathway.

## 1 INTRODUCTION

Psoriasis is recognized as a genetically inherited spectrum of skin illness mediated by immune which is portrayed as well‐demarcated erythematous plaques with adherent silvery white scales clinically.^1^ The global incidence rate of psoriasis among adults is approximately 0.51% ~ 11.43%, which may impose considerable burden on the life quality of psoriasis patients.^2^ Topical treatment, targeted phototherapy, biological agents, and oral systemic medications remain primary treatment approaches for psoriasis.^3^ However, effective strategies to cure psoriasis remain unavailable. Currently, it is generally believed that the immune‐inflammatory cascade mediated by the immune cell function and the release of inflammatory factors (e.g., tumor necrosis factor‐alpha [TNF‐α], interleukin‐1beta [IL‐1β], IL‐23, and IL‐12) are closely implicated in the onset and progression of psoriasis despite its inexact pathogenesis.^4^, ^5^ Therefore, the development of anti‐inflammatory agents may represent an effective therapeutic measure for psoriasis.

Chinese herbal medicine is well established as an ideal synergistic medicine applied to the diversified human diseases attributed to reduced adverse effects.^6^ Chinese herbal medicine has been increasingly revealed to exhibit effectiveness and safety as an adjuvant treatment of psoriasis.^7^, ^8^ *Scutellaria baicalensis* Georgi is a common heat‐clearing medicine in traditional Chinese medicine, the preparation from the root of which is widely applied to treat diarrhea, dysentery, hypertension, hemorrhaging, insomnia, inflammation, and respiratory infections in clinical settings.^9^ However, as a naturally occurring flavonoid compound that stems from the root extract of *S. baicalensis* Georgi, Wogonin has not been used in Western medicine in the form of a pure chemical. Wogonin has been supported to participate in tumors, inflammatory diseases, neurological diseases dependent on a wide range of pharmacological properties including antioxidant, anti‐inflammatory, antitumor, immunomodulatory as well as neuroprotective effects.^10^, ^11^ Notably, Wogonin has been underlined to suppress inflammatory cell infiltration and the production of pro‐inflammatory cytokines to produce protective effects against inflammatory disorders, such as mastitis,^12^ cerebral ischemia‐reperfusion injury,^13^ and so on. Moreover, previous literatures have introduced that Wogonin can ameliorate skin disorder through modulating the expressions of inflammation‐associated genes.^14^, ^15^ Nevertheless, whether Wogonin functions as an anti‐inflammatory agent in psoriasis remains to be elaborated.

Inflammasomes composed of multimeric protein complexes are sensors of molecular signals in response to infectious microbes and molecules derived from host proteins.^16^ As the most extensively studied inflammasome, nucleotide‐binding oligomerization domain (NOD)‐like receptor family pyrin domain containing 3 (NLRP3) inflammasome is a pivotal mediator in the immune response and disease development.^17^ Importantly, NLRP3 is highly expressed in psoriasis samples and the blockers of NLRP3 pathway may be recognized as novel therapeutic targets of psoriasis through ameliorating inflammatory reaction during the disease course.^18^, ^19^ Moreover, Wogonin has been supported to inactivate NLPP3 inflammasome in cerebral ischemia‐reperfusion injury.^13^

Here, our research was to illuminate the impacts of Wogonin on the progression of psoriasis and further delve into the mechanism of Wogonin related to NLRP3 inflammasome.

## 2 MATERIALS AND METHODS

### 2.1 Cell culture and treatment

Immortalized human epidermal cell line HaCaT (SAIOS) was nurtured in Dulbecco's modified Eagle medium (BasalMedia) supplied with 10% fetal bovine serum (BasalMedia) under the atmosphere of 5% CO_2_ at 37°C. Following the construction of psoriasis‐like keratinocytes model via exposure to M5 cytokines (TNF‐a, IL‐1α, IL‐17A, IL‐22, and oncostatin M, each at 2.5 ng/mL; PeproTech),^20^ HaCaT cells were treated by varying concentrations (4, 8, and 16 μM) of Wogonin (TargetMol) for 6 h.^21^

### 2.2 Plasmid transfection

The pcDNA3.1 plasmid (Addgene) carrying full‐length NLRP3 (Ov‐NLRP3) and the empty vector overexpression negative control (Ov‐NC) were transfected into HaCaT cells with Lipofectamine 3000 reagent (Invitrogen) as per the protocol of the manufacturer. After 48 h of transfection, cells were harvested.

### 2.3 Cell counting kit‐8 (CCK‐8)

In brief, before treatment with 50, 100, and 200 ng/mL of Wogonin with or without incubation with M5 cocktail, HaCaT cells were placed in a 96‐well plate (1 × 10^4^ cells/well), each well of which was then given 10‐μL CCK‐8 solution (Shanghai SunBio Medical Biotechnology Co. Ltd.) for 2 h in conformity to the standard manual. A microplate reader (Molecular Devices) was employed for the determination of OD450 nm value.

### 2.4 5‐Ethynyl‐2′‐deoxyuridine (EDU) staining

Cell proliferation was measured via the employment of EDU Cell Proliferation Image Kit (Abbkine). HaCaT cells (5 × 10^3^ cells/well) subjected to 96‐well plates were incubated with M5 cocktail and received treatment with 50, 100, and 200 ng/mL of Wogonin, before being labeled with 50‐µM EDU for 2 h in compliance with the manufacturer's request. Following the 15 min of immobilization with 4% paraformaldehyde and 10 min of permeation with 0.3% Triton X‐100, cells were conjugated to Click Reaction Buffer and Hoechst 33342. Images were taken under a fluorescence microscope (Olympus).

### 2.5 Enzyme‐linked immunosorbent assay (ELISA)

In the cell supernatants harvested by 10 min of centrifugation at 1000× g, TNF‐α (cat. no. ml077385), IL‐1β (cat. no. ml058059), IL‐6 (cat. no. ml058097), IL‐23 (cat. no. ml058067), IL‐17 (cat. no. ml058051), IL‐22 (cat. no. ml058066), C‐X‐C motif chemokine ligand 1 (CXCL1; cat. no. ml057794), C‐X‐C motif chemokine ligand 8 (CXCL8; cat. no. ml028580), and cysteine–cysteine motif chemokine ligand 20 (CCL20; cat. no. ml060006) levels were tested by related ELISA kits (Mlbio) in accordance with the manufacturer's guidance. Absorbance was tested using a microplate reader at 450 nm.

### 2.6 Immunofluorescence staining

In short words, 1% bovine serum albumin (BSA) was added to the HaCaT cells subjected to 15 min of soak in 4% paraformaldehyde at 4°C and 10 min of permeabilization with 0.1% Triton X‐100 for 1 h of blockade. Then, the cells were cultivated with NLRP3 (cat. no. 102161‐T02; 1/300; SinoBiological) and Caspase‐1 (cat. no. AF5418; 1/100; Affinity Biosciences) antibodies overnight at 4°C as well as Alexa Fluor‐488 conjugated goat anti‐rabbit IgG (cat. no. ab150077; 1/200; Abcam) for another 1 h at room temperature the following day. The visualization of nuclei was allowed via 10 min of staining with 1 mg/mL 4',6‐diamidinyl‐2‐phenylindole. Images were taken under a fluorescence microscope.

### 2.7 Reverse transcription‐quantitative PCR (RT‐qPCR)

The extraction of total RNA from HaCaT cells was accomplished with the employment of Trizol reagent (Absin). PCR amplification was carried out with synergy brands High‐Sensitivity qPCR SuperMix (Absin), referring to complementary DNA (cDNA) that was produced from RNA by reverse transcription using first Strand cDNA Synthesis SuperMix (Absin) as a template. Relative NLRP3 expression was calibrated in terms of 2^‐ΔΔCq^ approach. Glyceraldehyde‐3‐phosphate dehydrogenase (GAPDH) functioned as a normalization gene. The primer sequences were as follows: NLRP3, (forward) 5'‐ GATCTTCGCTGCGATCAACAG‐3′ and (reverse) 5′‐CGTGCATTATCTGAACCCCAC‐3′.

### 2.8 Western blot

After the preparation of cellular lysates by radioimmunoprecipitation assay buffer (Absin), the quantification of protein concentration was performed with the application of BCA method (Absin). Impeded by 5% BSA, the membranes that were to transfer proteins running on sodium dodecyl sulfate‐polyacrylamide gel electrophoresis were then labeled by keratin 6 (KRT6; cat. no. AG5345, 1/1000; Beyotime Biotechnology), cyclooxygenase‐2 (Cox2; cat. no. ab179800, 1/1000; Abcam), inducible nitric oxide synthase (iNOS; cat. no. ab283655, 1/1000; Abcam), NLRP3 (cat. no. ab263899, 1/1000; Abcam), apoptosis‐associated speck‐like protein containing a caspase recruit domain (ASC; cat. no. ab151700, 1/1000; Abcam), Gasdermin‐D (GSDMD; cat. no. ab219800, 1/1000; Abcam), GSDMD‐N‐terminal domain (GSDMD‐N; cat. no. #DF12275, 1/1000; Affinity Biosciences), Cleaved‐Caspase‐1 (cat. no. #AF4005, 1/1000; Affinity Biosciences), Caspase‐1 (cat. no. ab207802, 1/1000; Abcam), and GAPDH (cat. no. ab181603; 1/10000; Abcam) primary antibodies at 4°C overnight, before being probed with goat anti‐rabbit secondary antibody conjugated with HRP (ab6721; 1/2000; Abcam). The visualization of the blots was done by the electrochemiluminescence luminescence reagent (Shanghai Macklin Biochemical Co. Ltd.) and the gray analysis was implemented with ImageLab4.0 software (BioRad).

### 2.9 Statistical analyses

GraphPad Prism 8 software (GraphPad Software, Inc.) was utilized to carry out statistical analyses. All data from three parallel repeated experiments were denoted as the mean ± standard deviation. Statistical significances were measured by using of one‐way ANOVA along with Tukey's post hoc test. The significance level was *p* < 0.05.

## 3 RESULTS

### 3.1 Wogonin treatment obstructs the hyperproliferation of HaCaT cells exposed to M5

**Figure 1:**
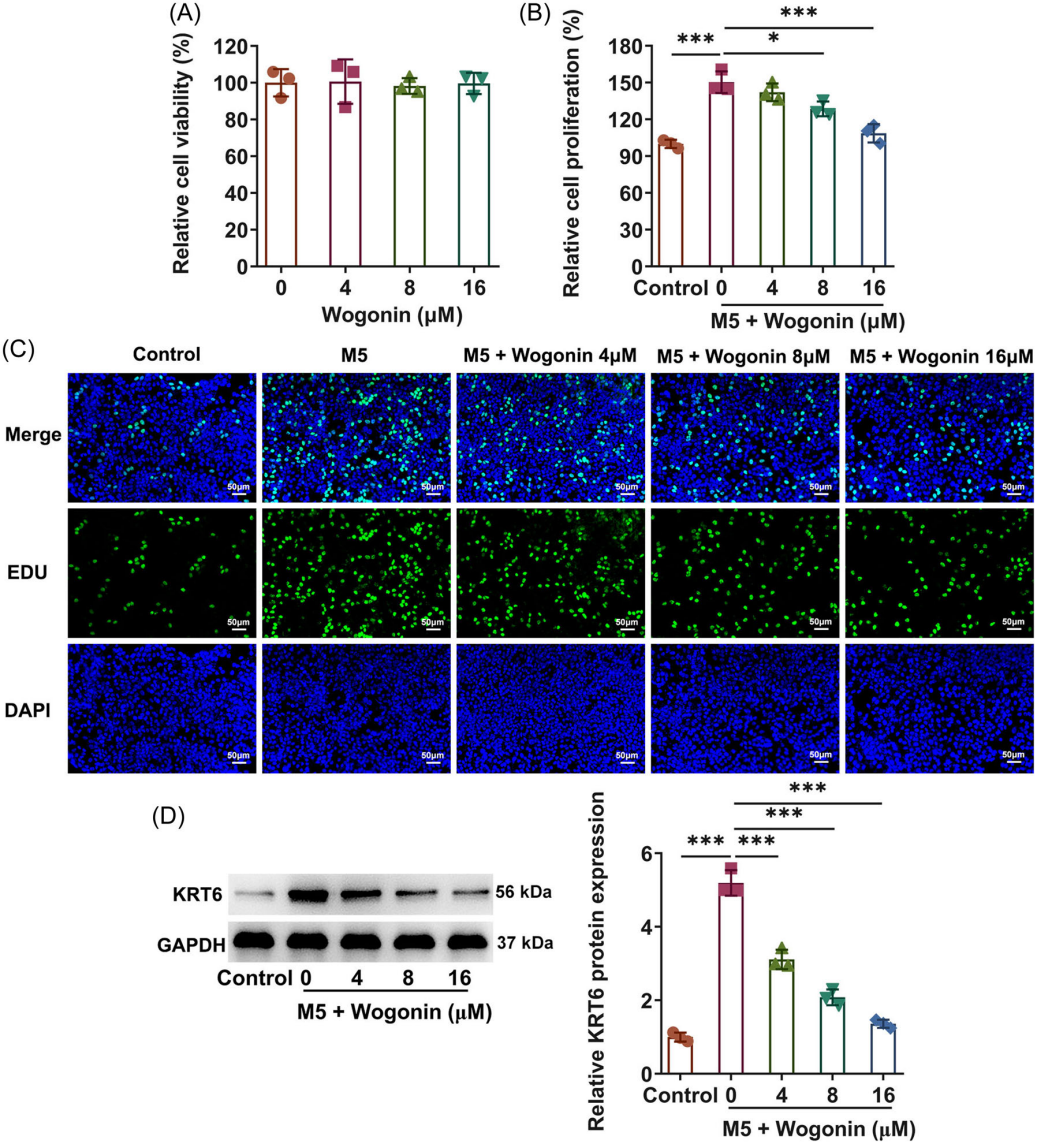
Wogonin treatment obstructs the hyperproliferation of HaCaT cells exposed to M5. (A) and (B) Cell counting kit‐8 method assayed cell viability. (C) 5‐Ethynyl‐2′‐deoxyuridine (EDU) staining measured cell proliferation. Magnification, ×200. (D) Western blot examined keratin 6 (KRT6) expression. The experiments were independently repeated in triplicate. The measurement data are expressed as mean ± SD. *p < 0.05 and ***p < 0.001. SD, standard deviation.

To identify the role of Wogonin in M5‐stimulated cellular model of psoriasis in HaCaT cells, the impacts of single Wogonin treatment on the viability of HaCaT cells were first estimated and it was noticed that HaCaT cell viability displayed no evident alternations upon treatment with increasing concentrations of Wogonin (4, 8, and 16 μM) (Figure 1A). Concurrently, as delineated by CCK‐8 assay, the viability of HaCaT cells was distinctly improved when exposed to M5, which was then concentration‐dependently diminished by Wogonin treatment (Figure 1B). Additionally, the experimental data from EDU staining presented that the potentiated proliferation of M5‐challenged HaCaT cells was halted by treatment with Wogonin in a dose‐dependent manner (Figure 1C). Western blot analysis also manifested that Wogonin markedly depleted KRT6 expression that was raised in M5‐treated HaCaT cells (Figure 1D). In summary, Wogonin suppressed M5‐induced HaCaT cell hyperproliferation.

### 3.2 Wogonin treatment mitigates the inflammatory response in HaCaT cells exposed to M5

**Figure 2:**
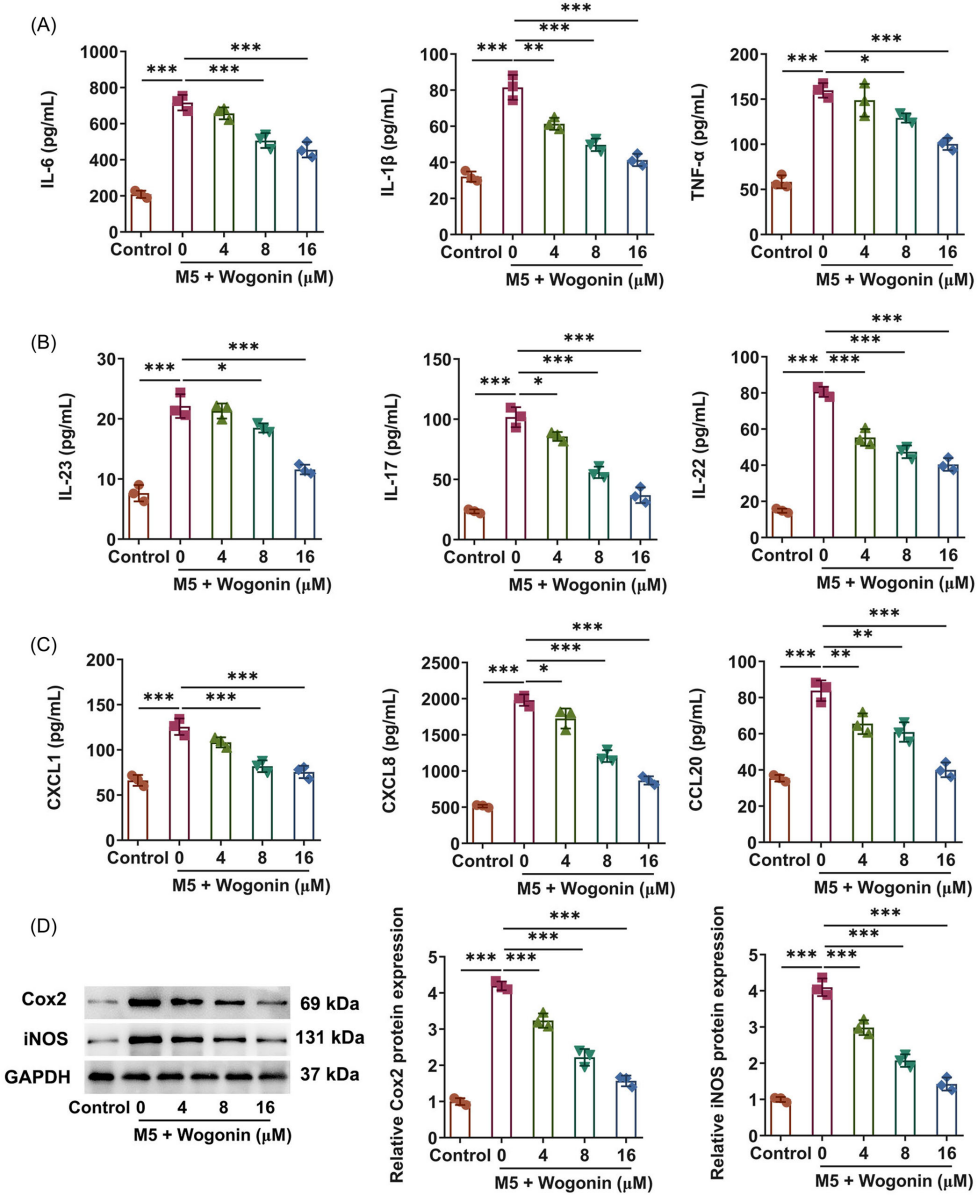
Wogonin treatment mitigates the inflammatory response in HaCaT cells exposed to M5. (A)–(C) enzyme‐linked immunosorbent assay analyzed the contents of inflammatory factors. (D) Western blot tested the expressions of inflammation‐related proteins. The experiments were independently repeated in triplicate. The measurement data are expressed as mean ± SD. *p < 0.05, **p < 0.01, and ***p < 0.001. CCL20, cysteine‐cysteine motif chemokine ligand 20; Cox2, cyclooxygenase‐2; CXCL1, C‐X‐C motif chemokine ligand 1; CXCL8, C‐X‐C motif chemokine ligand 8; IL‐17, interleukin‐17; IL‐1β, interleukin‐1beta; IL‐22, interleukin‐22; IL‐23, interleukin‐23; IL‐6, interleukin‐6; iNOS, inducible nitric oxide synthase; TNF‐α, tumor necrosis factor‐alpha.

Inflammatory response remains a crucial event during the process of psoriasis. Subsequently, the effects of Wogonin on the inflammatory response were assessed in M5‐induced psoriasis model in vitro. Through ELISA analysis, it turned out that M5 exposure resulted in the remarkable upregulation on the contents of inflammatory cytokines including IL‐6, TNF‐α, and IL‐1β in HaCaT cells, which were then all declined by Wogonin (Figure 2A). As expected, in M5‐challenged HaCaT cells, the elevated activities of inflammatory molecules that emerged as critical components in the pathogenesis of psoriasis including IL‐23, IL‐17, and IL‐22 were all prominently lowered by Wogonin, exhibiting a concentration‐dependent manner (Figure 2B). It was also discovered that Wogonin dramatically lessened the levels of pro‐inflammatory chemokines including CXCL1, CXCL8, and CCL20 that were enhanced in HaCaT cells imposed by M5 exposure (Figure 2C). Also, the expressions of inflammatory mediators Cox2 and iNOS were noted to be augmented in M5‐exposed HaCaT cells and be downregulated in Wogonin‐treated HaCaT cells challenged with M5 (Figure 2D). Accordingly, Wogonin might protect against M5‐triggered inflammatory response in HaCaT cells.

### 3.3 Wogonin treatment inactivates NLRP3/Caspase‐1/GSDMD pyroptosis pathway in HaCaT cells exposed to M5

**Figure 3:**
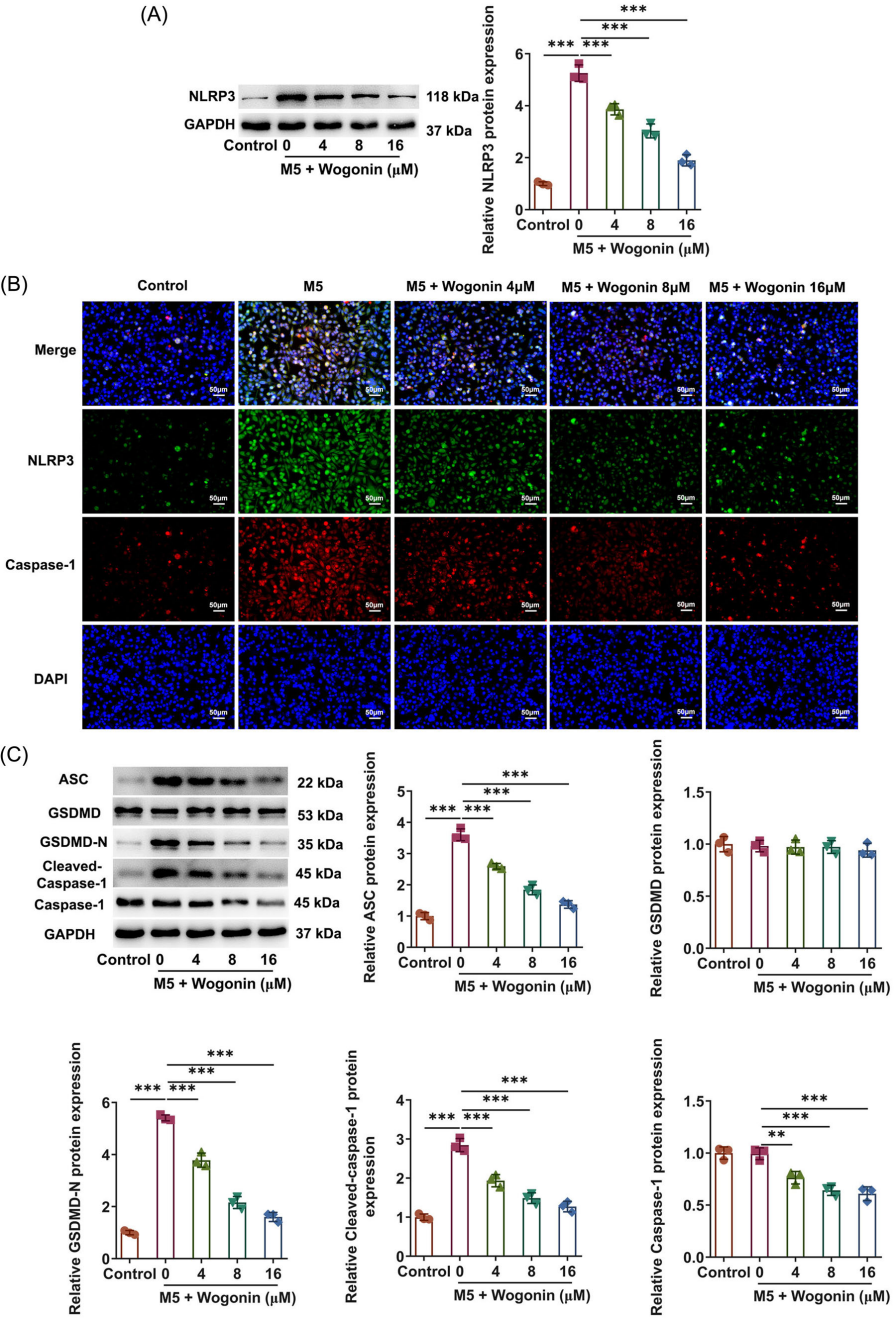
Wogonin treatment inactivates NLRP3/Caspase‐1/GSDMD pyroptosis pathway in HaCaT cells exposed to M5. (A) Western blot examined NLRP3 expression. (B) Immunofluorescence staining detected NLRP3 and Caspase‐1 expressions. Magnification, ×200. (C) Western blot tested the expressions of pyroptosis‐related proteins. The experiments were independently repeated in triplicate. The measurement data are expressed as mean ± SD. **p < 0.01 and ***p < 0.001. ASC, apoptosis‐associated speck‐like protein containing a caspase recruit domain; GSDMD, Gasdermin‐D; GSDMD‐N, GSDMD‐N‐terminal domain; NLRP3, NOD‐like receptor family pyrin domain containing 3; NOD, nucleotide‐binding oligomerization domain; SD, standard deviation.

Pyroptosis mediated by NLRP3/Caspase‐1/GSDMD pathway has been viewed as a participator in psoriasis. Through Western blot and immunofluorescence staining, it was observed that the ascending NLRP3 and Caspase‐1 expressions in M5‐challenged HaCaT cells both presented the downward trend by Wogonin (Figure 3A,B). Besides, the expressions of pyroptosis‐associated proteins were examined with Western blot and the results elucidated that M5 induction greatly fortified ASC, GSDMD‐N, and Cleaved‐Caspase‐1 expressions in HaCaT cells and Wogonin concentration‐dependently decreased ASC, GSDMD‐N, Cleaved‐Caspase‐1, and Caspase‐1 expressions in HaCaT cells upon exposure to M5 (Figure 3C). Overall, Wogonin hindered M5‐stimualted HaCaT cell pyroptosis mediated by NLRP3/Caspase‐1/GSDMD pathway.

### 3.4 Wogonin inactivates NLRP3 to suppress M5‐elicited hyperproliferation of HaCaT cells

**Figure 4:**
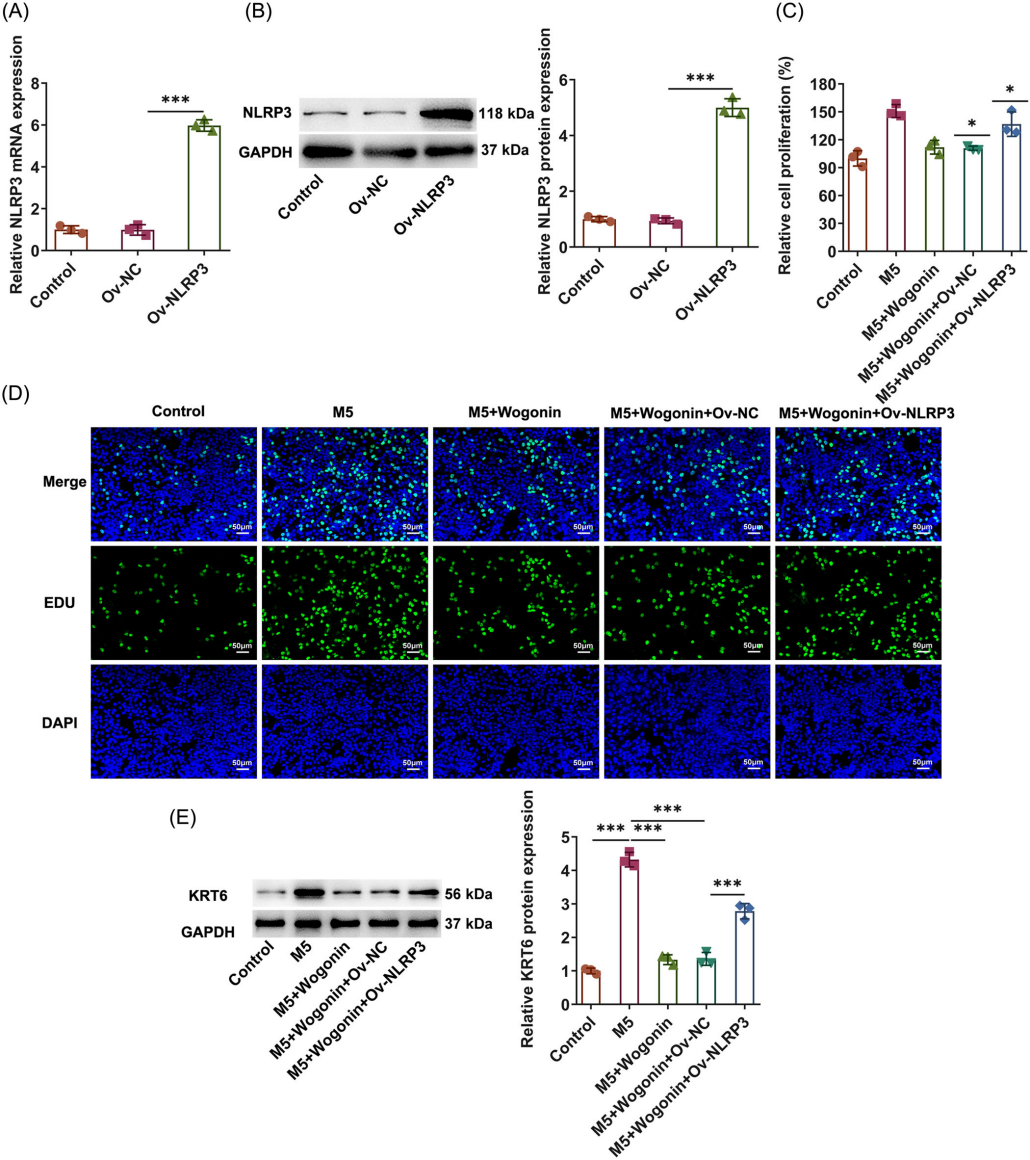
Wogonin inactivates NLRP3 to suppress M5‐elicited hyperproliferation of HaCaT cells. (A) Reverse transcription‐quantitative PCR and (B) Western blot examined the transduction efficacy of NLRP3 overexpression plasmids. (C) Cell counting kit‐8 method assayed cell viability. (D) 5‐Ethynyl‐2′‐deoxyuridine (EDU) staining measured cell proliferation. Magnification, ×200. (E) Western blot examined KRT6 expression. The experiments were independently repeated in triplicate. The measurement data are expressed as mean ± SD. *p < 0.05, **p < 0.01, and ***p < 0.001. NLRP3, NOD‐like receptor family pyrin domain containing 3; NOD, nucleotide‐binding oligomerization domain; KRT6, keratin 6; SD, standard deviation.

To determine whether the possible regulatory mechanism of Wogonin in psoriasis was associated with NLRP3/Caspase‐1/GSDMD‐mediated pyroptosis, NLRP3 was overexpressed. Following transfection of Ov‐NLRP3, NLRP3 messenger RNA, and protein expressions were both distinctly increased in HaCaT cells (Figure 4A,B). CCK‐8 and EDU assays corroborated that the inhibitory role of Wogonin in the proliferation of M5‐treated HaCaT cells was partially abolished when NLRP3 was upregulated (Figure 4C,D). This finding was also further testified by the raised KRT6 expression in the M5 + Wogonin + Ov‐NLRP3 group relative to the M5 + Wogonin + Ov‐NC group (Figure 4E). Conclusively, NLRP3 elevation partially reversed the influences of Wogonin on the hyperproliferation of M5‐exposed HaCaT cells.

### 3.5 Wogonin inactivates NLRP3 to protect against M5‐elicited inflammatory response in HaCaT cells

**Figure 5:**
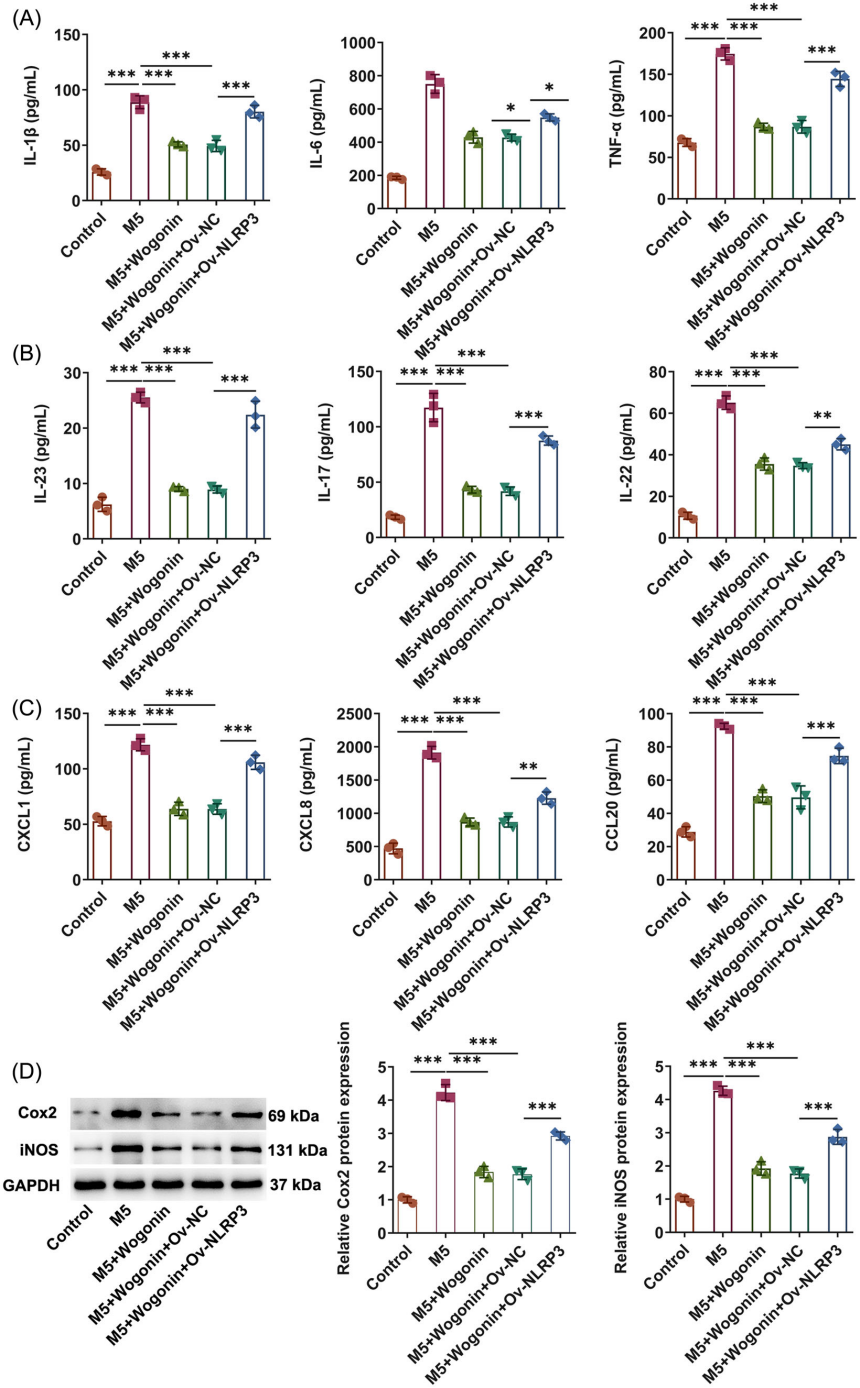
Wogonin inactivates NLRP3 to protect against M5‐elicited inflammatory response in HaCaT cells. (A)–(C) Enzyme‐linked immunosorbent analyzed the contents of inflammatory factors. (D) Western blot tested the expressions of inflammation‐related proteins. The experiments were independently repeated in triplicate. The measurement data are expressed as mean ± SD. **p < 0.01 and ***p < 0.001. CCL20, cysteine‐cysteine motif chemokine ligand 20; Cox2, cyclooxygenase‐2; CXCL1, C‐X‐C motif chemokine ligand 1; CXCL8, C‐X‐C motif chemokine ligand 8; IL‐17, interleukin‐17; IL‐1β, interleukin‐1beta; IL‐22, interleukin‐22; IL‐23, interleukin‐23; IL‐6, interleukin‐6; iNOS, inducible nitric oxide synthase; NLRP3, NOD‐like receptor family pyrin domain containing 3; NOD, nucleotide‐binding oligomerization domain; SD, standard deviation; TNF‐α, tumor necrosis factor‐alpha.

At the same time, in M5‐exposed HaCaT cells, Wogonin obviously diminished IL‐6, TNF‐α, IL‐1β, IL‐23, IL‐17, IL‐22, CXCL1, CXCL8, and CCL20 activities, which were then elevated again after NLRP3 was overexpressed (Figure 5A–C). In addition, NLRP3 fortified Cox2 and iNOS expressions that were depleted in Wogonin‐treated HaCaT cells challenged with M5 (Figure 5D). All these results pointed out that NLRP3 elevation partially reversed the influences of Wogonin on the inflammatory response in M5‐exposed HaCaT cells.

### 3.6 Wogonin inactivates NLRP3 to hamper M5‐elicited pyroptosis in HaCaT cells

**Figure 6:**
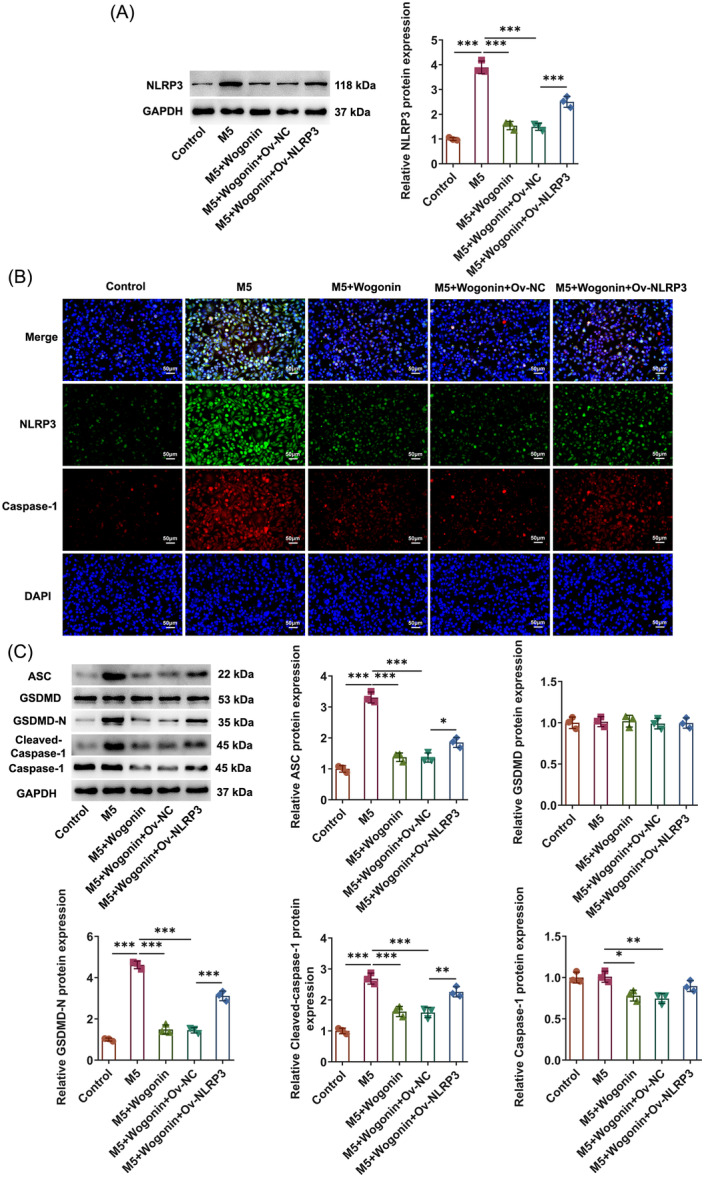
Wogonin inactivates NLRP3 to hamper M5‐elicited pyroptosis in HaCaT cells. (A) Western blot examined NLRP3 expression. (B) Immunofluorescence staining detected NLRP3 and Caspase‐1 expressions. Magnification, ×200. (C) Western blot tested the expressions of pyroptosis‐related proteins. The experiments were independently repeated in triplicate. The measurement data are expressed as mean ± SD. *p < 0.05 and ***p < 0.001. ASC, apoptosis‐associated speck‐like protein containing a caspase recruit domain; GSDMD, Gasdermin‐D; GSDMD‐N, GSDMD‐N‐terminal domain; NLRP3, NOD‐like receptor family pyrin domain containing 3; NOD, nucleotide‐binding oligomerization domain.

Further, Wogonin conspicuously inhibited NLRP3 and Caspase‐1 expressions in HaCaT cells treated by M5 and NLRP3 elevation improved NLRP3 and Caspase‐1 expressions in Wogonin‐treated HaCaT cells upon exposure to M5 (Figure 6A,B). Also, ASC, GSDMD‐N, Cleaved‐Caspase‐1, and Caspase‐1 expressions were declined in M5 + Wogonin relative to M5 group. By contrast with M5 + Wogonin + Ov‐NC group, upregulation of NLRP3 motivated ASC, GSDMD‐N, and Cleaved‐Caspase‐1 expressions in M5 +  Wogonin + Ov‐NLRP3 group (Figure 6C). Taken together, NLRP3 elevation partially reversed the influences of Wogonin on the NLRP3/Caspase‐1/GSDMD pathway‐mediated pyroptosis in M5‐exposed HaCaT cells.

## 4 DISCUSSION

Psoriasis is a systemic disease accompanied with complicated pathogenic mechanisms involving inflammatory disorder, genetic effects, and activated immune response. As the predominant cell type in the epidermis, keratinocytes are responsible for the formation of a physical skin barrier against environmental damages through producing antimicrobial peptides and cytokines and further attracting and activating immune cells to participate in the inflammatory mechanism in the lesions, thus playing an active role in the formation and maintenance of psoriatic lesions.^22^, ^23^ When the epidermal barrier of psoriasis is destructed, keratinocytes are susceptible to diverse external harmful substances, causing cell damage or even cell death.^23^ Accordingly, the abnormal interaction between hyperproliferative epidermal keratinocytes and self‐reactive immune cells is commonly known as a hallmark of psoriasis.^22^ HaCaT cells are immortalized keratinocyte cell lines widely used in scientific research. Thereafter, HaCaT cells were induced by M5 to assess aberrant keratinocyte behaviors in psoriasis. Wogonin has been previously revealed to hamper cell proliferation and invasion in skin epithelioid carcinoma^24^ and exhibit potent protective properties against skin damage.^14^, ^15^, ^25^, ^26^ However, the exact role of Wogonin in psoriasis is unclear. In the present study, HaCaT cells were exposed to M5 cytokines to mimic psoriasis in vitro. Administration with Wogonin eliminated the hyperproliferation, inflammatory response as well as pyroptosis in M5‐stimulated psoriatic keratinocyte model, the protective mechanism of which might be mediated by inactivation of NLRP3.

As reported, traditional Chinese medicine *S. baicalensis* Georgi can ameliorate the macrophage‐targeted responses and arrest keratinocyte proliferation to exert pharmaceutical efficacy in psoriasis‐like lesions.^27^ In particular, Wang et al have disclosed that Wogonin, a main effective component of *S. baicalensis* Georgi, can reduce photodamage in HaCaT cells induced by ultraviolet B radiation.^28^ Here, the current study demonstrated that the improved viability and proliferation of HaCaT cells challenged with M5 were both diminished by Wogonin treatment in a concentration‐dependent manner. KRT6 is a member of Keratins that are known as the major components of the epithelial cytoskeleton associated with the maintenance of the structural stability and integrity of keratinocytes.^29^ Also, this research hinted that Wogonin downregulated the expression of KRT6, a marker for hyperplasia, in M5‐treated HaCaT cells. Inflammatory response remains a crucial event in the initiation and progression of psoriasis.^30^ Besides, the hyperproliferative keratinocytes sustain and amplify the inflammatory response via expressing cytokines.^31^ TNF‐α, IL‐1β, IL‐23, IL‐17, IL‐22, Cox2, and iNOS are all inflammatory cytokines involved in the pathogenesis of psoriasis,^32^, ^33^ and CXCL1, CXCL8, and CCL20 are antimicrobial peptides and chemokines produced by keratinocytes.^34^ Notably, Wogonin has been previously unraveled to suppress pro‐inflammatory Cox2, iNOS, TNF‐α, and IL‐1β expression in skin diseases.^14^, ^15^, ^25^, ^35^ Consistently, the present experimental data proved that M5 exposure resulted in the upregulation on the contents of inflammatory cytokines including IL‐6, TNF‐α, IL‐1β, IL‐23, IL‐17, IL‐22 CXCL1, CXCL8, CCL20, and the expression of Cox2, iNOS in HaCaT cells, which were then all concentration‐dependently declined by Wogonin.

Notably, Wogonin has been introduced to block NLRP3 inflammasome activation which emerges as a key mediator of psoriatic inflammation.^13^, ^18^, ^36^ It is well documented that the activation of NLRP3 inflammasome is essential for pyroptosis in various diseases, psoriasis is also included.^37^ Upon activation by a wide range of stimuli, the innate immune sensor protein NLRP3 oligomerizes and interacts with adaptor protein ASC, then recruits caspase‐1 to drive the assembly of NLRP3 inflammasome, resulting in the cleavage and activation of caspase‐1 in turn and further cleaving GSDMD and releasing the cleaved GSDMD‐N to form membrane pore, thereby contributing to pyroptosis.^38^ Moreover, Wogonoside can block GSDMD‐mediated pyroptosis to suppress cisplatin‐stimulated cardiotoxicity.^39^ In addition, Zhou et al. have supported that Wogonoside hampers pyroptosis to protect against obesity‐elicited lipid metabolism disorders and cardiac injury.^40^ In this study, it turned out that NLRP3, Caspase‐1, ASC, GSDMD‐N, and Cleaved‐Caspase‐1 expressions were both augmented in HaCaT cells upon exposure to M5, which were all depleted on account of treatment with Wogonin. Further elevation of NLRP3 partially counteracted the inhibitory role of Wogonin in M5‐triggered hyperproliferation, inflammatory response, as well as pyroptosis in HaCaT cells.

Several limitations should be noted in this study. First, our study only preliminarily revealed the protective role of Wogonin in M5‐stimulated psoriatic keratinocyte model in vitro and the impacts of Wogonin on the severity of psoriatic response need to be further unveiled in vivo. Second, pharmacokinetic/pharmacodynamics analysis of Wogonin is lacking here, which needs to be further performed in the future. Third, whether Wogonin targets other possible molecular signaling pathways to participate in the process of psoriasis also warrants further investigation. Besides, the possible receptors of Wogonin on HaCaT cells also need to be identified in our future study.

## 5 CONCLUSION

All in all, Wogonin was capable of alleviating the hyperproliferation, inflammatory response as well as pyroptosis in HaCaT keratinocytes exposed to M5 cytokines, the effects of which might be related to the blockade of NLRP3/Caspase‐1/GSDMD pathway. This finding implied that Wogonin might serve as an attractive candidate for future development as an anti‐psoriatic agent.

## AUTHOR CONTRIBUTIONS

**Jun Ma**: Conceptualization; investigation; methodology; validation; writing—original draft. **Chen Ji**: Conceptualization; funding acquisition; project administration; supervision. **Yanhong Sun**: Data curation; investigation; methodology; visualization. **Danqing Liu**: Software; visualization; writing—review and editing. **Kai Pan**: Methodology; writing—review and editing. **Yuegang Wei**: Conceptualization; funding acquisition; project administration.

## Data Availability

All data generated or analyzed during this study are included in this published article.
